# P-1739. Impact of a Urinary Tract Infection Treatment Algorithm on Antimicrobial Prescribing at a Small Rural Hospital without Formally Trained Infectious Disease Team Members

**DOI:** 10.1093/ofid/ofae631.1902

**Published:** 2025-01-29

**Authors:** Daniel West, Shane Becker, Nancy McClew

**Affiliations:** Grand River Health, Rifle, Colorado; Grand River Health, Rifle, Colorado; Grand River Health, Rifle, Colorado

## Abstract

**Background:**

Small, rural community hospitals lack certified infectious disease (ID) staff, which can make antimicrobial stewardship (AMS) interventions challenging. At our Critical Access Hospital, we developed a treatment algorithm for urinary tract infections (UTIs) and assessed appropriate duration of therapy (DOT), and appropriate antimicrobial selection in cases before and after implementation of our algorithm.

Figure 1.

**Figure:**
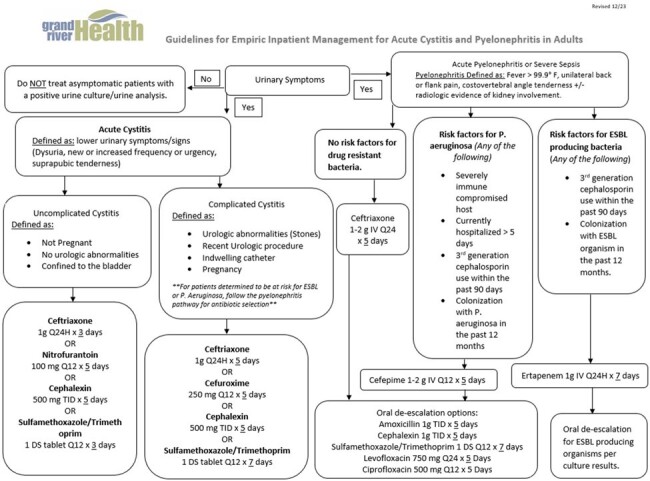

**Methods:**

In this retrospective observational study, the AMS team published a treatment algorithm for UTIs (Figure 1) in Q1 2023. Implementation of the algorithm focused on physician exposure, buy-in, and follow-up. A webpage was created to house the algorithm. Buy-in was obtained from key physicians. Antimicrobial prescribing was reviewed at quarterly AMS meetings and physician leaders took this information back to their teams for review. A retrospective review was performed after 2 quarters of interventions.

We obtained prescribing data via electronic health records. Baseline characteristics are in Table 1. Each UTI case was manually reviewed for DOT and appropriateness of antibiotic choice as defined by agreement with our algorithm.

**Figure:**
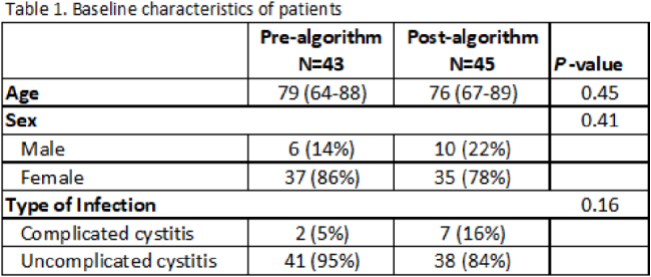

**Results:**

The study included in-patients who were treated for UTI between 10/1/21 through 3/31/22, and 10/1/23 through 3/31/24. Exclusion criteria included concomitant infections, inability to follow-up, and expiration. A t-test was performed to compare antibiotic DOT for UTI treatment. The distribution of DOT is presented in Figure 2. There was a decrease in DOT after our intervention (M=3.84, SD 1.95) compared to baseline (M=4.95, SD=2.64); t(2.2491), P=0.0271. Appropriateness of therapy was assessed (Figure 3). A Chi-square test showed association between the intervention and appropriate choice of therapy, χ2 (1, N= 88)= 12.90, p=0.0003.

Figure 2.

**Figure:**
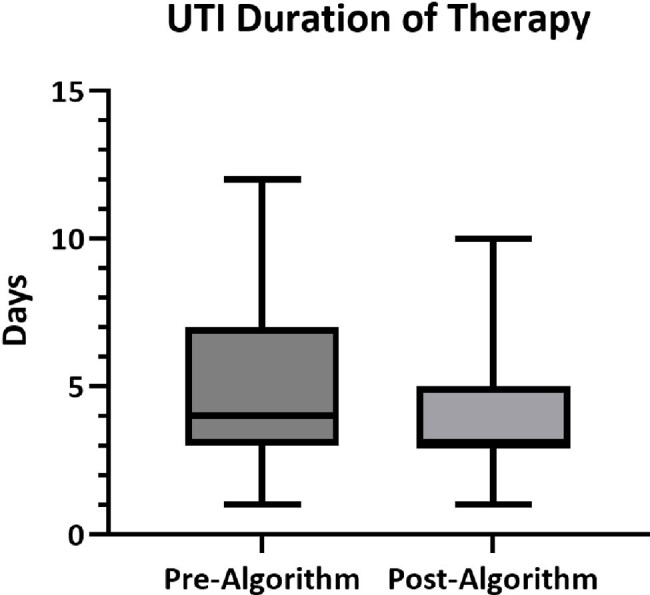

Antibiotic days for the treatment of UTI. Antibiotic days calculated to include discharge medications.

**Conclusion:**

The development of a treatment algorithm for UTI, paired with stakeholder buy-in, exposure, and follow-up, led to shifts in prescribing habits. We found a reduction in DOT, as well as better adherence to guideline driven antibiotic selection. This study, which included discharge prescribing, is unique in that it follows our cases through the continuum of care. We show that rural hospitals without certified ID staff can effectively implement AMS best practices and affect meaningful change.

Figure 3.

**Figure:**
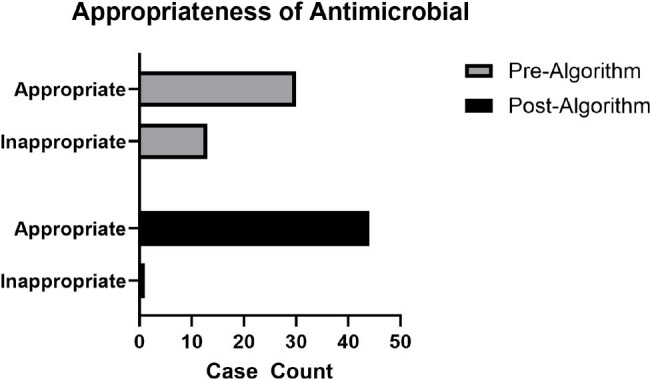

Retrospective contingency table summarizing number of appropriate and inappropriately treated UTIs. Appropriateness determined by AMS team by comparing antibiotic selection to our UTI algorithm.

**Disclosures:**

**All Authors**: No reported disclosures

